# Editorial: Diversity in stroke omic(s) and epidemiology research: opportunities and challenges

**DOI:** 10.3389/fstro.2024.1421083

**Published:** 2024-05-13

**Authors:** N. Abimbola Sunmonu, Keith L. Keene, Hyacinth I. Hyacinth

**Affiliations:** ^1^Department of Neurology, Yale University School of Medicine, New Haven, CT, United States; ^2^Center for Health Equity and Precision Public Health, University of Virginia Health System, Charlottesville, VA, United States; ^3^Department of Neurology and Rehabilitation Medicine, University of Cincinnati College of Medicine, Cincinnati, OH, United States

**Keywords:** genomics, omics, stroke, health disparities, epidemiology

## Introduction

Stroke is the leading cause of disability and second leading cause of death worldwide (GBD 2019 Stroke Collaborators, 2021; Saini et al., 2021; Feigin et al., 2022). Globally, ~15 million people suffer a stroke annually: one-third die, another third are permanently disabled (World Health Organisation, 2024). Stroke costs >$721 billion annually worldwide in healthcare and missed productivity (Feigin et al., 2022). Dramatic health disparities exist in stroke risk, treatment and outcomes, with higher incidence, complications and fatality in people of non-European ancestry and lower socioeconomic status (SES; Fukino et al., 2007; Carty et al., 2015; Carnethon et al., 2017; Kamin Mukaz et al., 2020; Chandler et al., 2021; Akam et al., 2022). Lower income countries have almost 90% of stroke deaths and disability (Feigin et al., 2022). Translating knowledge from stroke genetics is challenging in part because of the dearth of diverse studies −95% of large genomic studies and clinical trials include solely European ancestry populations (Mills and Rahal, 2020). Furthermore, there is limited genetic testing, particularly in the socioeconomic and racial groups most impacted (Carty et al., 2015; Carnethon et al., 2017; Ferrario et al., 2017; Kamin Mukaz et al., 2020; Brown et al., 2021; Prapiadou et al., 2021; Denorme et al., 2023). This Research Topic, entitled “*Diversity in stroke omic(s) and epidemiology research: opportunities and challenges*,” features five original papers as examples of multifaceted approaches to exploring diversity in the populations, clinically relevant problems and techniques in stroke genomics research. They include an exploration of population needs assessments to identify potential gaps in health literacy, and a roadmap for critical community engagement necessary to translate genomic knowledge into clinical practice and social policies.

First, Brown et al. examine underrepresentation in genomic medicine, especially for stroke. Using Hawai'i and the Pacific Islands as a case study, they highlight important consequences of underrepresentation and suggest avenues to improve participation of diverse populations in stroke genomic studies. Distinguishing between racial identity, a social construct, and genetic markers of ancestry, the authors reinforce our knowledge that perpetuating current exclusionary practices (intentional or unintentional) in genomic studies will result in development of biased protocols and practices that do not apply to non-European ancestry groups, and treatments from which they do not benefit. They argue that to successfully recruit diverse participants in genomic stroke studies, investigators must seek to understand their target communities, align research goals with the community's values, engage them early in study design, and prioritize their rights.

There is no cure for stroke. Only two treatments for acute stroke exist: intravenous thrombolysis and endovascular thrombectomy. Most patients are ineligible for either because they seek medical attention too late or their causative lesion is not amenable to thrombectomy (O'Connor et al., 1999; de Los Ríos la Rosa et al., 2012). Early symptom recognition and receiving timely emergency medical care and acute treatments are critical. In their study of African American adults in South Carolina, the American state with the greatest stroke incidence and mortality, Sunmonu et al. show that two aspects of stroke literacy—knowledge of stroke signs and symptoms and intent to call emergency medical services (EMS) in the event of a stroke—were significantly lower in older adults, males, and those with high school education or less. Consistent with previous reports (Miller et al., 2007; Williams et al., 2012), greater awareness of stroke symptoms increased intent to call EMS in the event of a stroke. Encouragingly, stroke literacy and intent to call EMS were higher in stroke survivors and increased over the 18-year course of their study, highlighting the impact of stroke education on stroke survivors and perhaps the population overall. It also demonstrates population-specific opportunities to improve timely emergency room arrivals and therefore improve eligibility for acute stroke treatments.

Genomic studies can inform whether our understanding of biologic processes can be translated to different physiologic milieux. In a clinical study, Ruhl et al. show there are important nuances in different biological niches that may preclude findings from one being translated to another. For example, nitric oxide (NO) has a complex role in stroke hemodynamic regulation but overall appears to improve blood flow (Radomski et al., 1987, 1990). Alpha globin (HBA) regulates endothelial NO, and genetically deleted HBA appears to protect from kidney disease, presumably via increased NO. Since HBA copy number is variable in people of African and Asian ancestries, Ruhl et al. postulated that HBA copy number could mitigate stroke risk in these populations. However, they showed that incident ischemic stroke is independent of HBA copy number in a cohort of African ancestry individuals. Their findings reiterate that when ancestry-specific genomic information drives clinically relevant, hypothesis-driven studies, the resulting information can be quite different from that anticipated on the basis of existing studies and known physiology. This impacts the applicability of other extracranial vascular mechanisms to stroke risk.

Beyond well-known vascular risk factors (e.g., hypertension and diabetes), investigators continue searching for new clinical stroke risk factors to better define at-risk individuals. Here, Jayaraman et al. suggest pulmonary hypertension (PH) may be one such factor. In a cohort of hospitalized patients with incident stroke, most of them also had PH. Concurrent PH increased length of stay and stroke-related mortality. This effect was greater in patients who were younger, female, and Hispanic, Native American, Asian or Pacific Islander. Pulmonary hypertension and concurrent ischemic stroke occurred in Black patients almost a decade earlier than White patients. In all, their study supports PH as a risk factor for stroke, and its impact varies across populations.

Nearly one-third of strokes are recurrent events. People who suffer a recurrent stroke are more likely to become disabled or die (Skajaa et al., 2022; Aked et al., 2024). As with incident stroke, these outcomes are magnified in people of African ancestry compared with those of European ancestry (Park and Ovbiagele, 2016; Albright et al., 2018; Castello et al., 2021; Robinson et al., 2022). In a genome-wide association study (GWAS) using cohorts of diverse ancestral origin, Aldridge et al. examined the genetic risk of recurrent stroke. They discovered 18 unique genetic loci significantly associated with increased risk of recurrent stroke. These loci have known stroke biological relevance. Importantly, the authors also demonstrate that polygenic risk scores designed for incident stroke may not be applicable to recurrent stroke, suggesting that recurrent stroke may be a distinct phenotype from incident stroke.

## Summary and conclusions

This Research Topic demonstrates the importance of broadly diverse participants, methodologies and clinical questions in stroke genomics specifically and stroke epidemiology in general. Genomic studies can minimize stroke disparities by identifying unique genetic profiles to predict stroke risk and even guide customized therapeutics (Mishra et al., 2022). However, lack of participant diversity in current stroke genomics research effectively excludes underrepresented populations from potential and novel therapeutic interventions based on these studies and perhaps exposes them to unnecessary risk from such derived therapies. Genetically defined factors such as ancestry are important modifiers of stroke risk and should inform overall clinical risk assessment at the individual level for better, more accurate and personalized care. Enriching the current pool of genomic data with information from multi-ancestry cohorts is an important first step in ensuring broad representation in genomic reference databases and easier identification of key genomic factors that can accelerate the development of targeted therapies with great potential to benefit the general population.

A huge knowledge gap exists in our translation of genomic predictors of stroke risk into prevention and treatments for at-risk populations. Early identification of genetically vulnerable individuals could have a seismic impact, similar to the paradigm already implemented in cancer therapeutics. Additional screening for validated genetic and genomic variants from representative populations may complement clinical stroke screening. Despite the current challenges of genomic stroke studies, opportunities abound to leverage advanced genomic technologies to investigate novel dimensions of stroke risk in order to minimize incidence, mitigate severity and enhance recovery.

## Author contributions

NS: Writing – original draft, Writing – review & editing. KK: Writing – review & editing. HH: Writing – review & editing.

## References

[B1] Akam E. Y. Nuako A. A. Daniel A. K. Stanford F. C. (2022). Racial disparities and cardiometabolic risk: new horizons of intervention and prevention. Curr. Diab. Rep. 22, 129–136. 10.1007/s11892-022-01451-6 35175453 PMC9908372

[B2] Aked J. Delavaran H. Wennerström F. Lindgren A. G. (2024). Recovery, functional status, and health-related quality of life status up to 4 years after first ever stroke onset. A population-based study. Neuroepidemiology 2024:538222. 10.1159/000538222 38531332 PMC11633868

[B3] Albright K. C. Huang L. Blackburn J. Howard G. Mullen M. Bittner V.. (2018). Racial differences in recurrent ischemic stroke risk and recurrent stroke case fatality. Neurology 91, e1741–e1750. 10.1212/WNL.0000000000006467 30282770 PMC6251602

[B4] Brown L. L. Zhang Y. S. Mitchell U. (2021). Black older adults in the age of biomarkers, physical functioning, and genomics: heterogeneity, community engagement, and bioethics. Annu. Rev. Gerontol. Geriatr. 41, 183–210. 37008388 PMC10065475

[B5] Carnethon M. R. Pu J. Howard G. Albert M. A. Anderson C. A. M. Bertoni A. G.. (2017). Cardiovascular health in African Americans: a scientific statement from the American Heart Association. Circulation 136, e393–e423. 10.1161/CIR.0000000000000534 29061565

[B6] Carty C. L. Keene K. L. Cheng Y. C. Meschia J. F. Chen W. M. Nalls M.. (2015). Meta-analysis of genome-wide association studies identifies genetic risk factors for stroke in African Americans. Stroke 46, 2063–2068. 10.1161/STROKEAHA.115.009044 26089329 PMC4740911

[B7] Castello J. P. Pasi M. Abramson J. R. Rodriguez-Torres A. Marini S. Demel S.. (2021). Contribution of racial and ethnic differences in cerebral small vessel disease subtype and burden to risk of cerebral hemorrhage recurrence. Neurology 96, e2469–e2480. 10.1212/WNL.0000000000011932 33883240 PMC8205476

[B8] Chandler P. D. Clark C. R. Zhou G. Noel N. L. Achilike C. Mendez L.. (2021). Hypertension prevalence in the All of Us Research Program among groups traditionally underrepresented in medical research. Sci. Rep. 11:12849. 10.1038/s41598-021-92143-w 34158555 PMC8219813

[B9] de Los Ríos la Rosa F. Khoury J. Kissela B. M. Flaherty M. L. Alwell K. Moomaw C. J.. (2012). Eligibility for intravenous recombinant tissue-type plasminogen activator within a population: the effect of the European Cooperative Acute Stroke Study (ECASS) III trial. Stroke 43, 1591–1595. 10.1161/STROKEAHA.111.645986 22442174 PMC3361593

[B10] Denorme F. Armstrong N. D. Stoller M. L. Portier I. Tugolukova E. A. Tanner R. M.. (2023). The predominant PAR4 variant in individuals of African ancestry worsens murine and human stroke outcomes. J. Clin. Invest. 133:1169608. 10.1172/JCI169608 37471144 PMC10503801

[B11] Feigin V. L. Brainin M. Norrving B. Martins S. Sacco R. L. Hacke W.. (2022). World Stroke Organization (WSO): global stroke fact sheet 2022. Int. J. Stroke 17, 18–29. 10.1177/17474930211065917 34986727

[B12] Ferrario M. M. Veronesi G. Kee F. Chambless L. E. Kuulasmaa K. Jørgensen T.. (2017). Determinants of social inequalities in stroke incidence across Europe: a collaborative analysis of 126,635 individuals from 48 cohort studies. J. Epidemiol. Community Health 71, 1210–1216. 10.1136/jech-2017-209728 28983063

[B13] Fukino C. L. Pobutsky A. Baker K. K. Tottori C. Salvail F. (2007). The Burden of Cardiovascular Disease in Hawaii 2007. Honolulu, HI: Hawaii State Department of Health.

[B14] GBD 2019 Stroke Collaborators (2021). Global, regional, and national burden of stroke and its risk factors, 1990-2019: a systematic analysis for the Global Burden of Disease Study 2019. Lancet Neurol. 20, 795–820. 10.1016/S1474-4422(21)00252-0 34487721 PMC8443449

[B15] Kamin Mukaz D. Zakai N. A. Cruz-Flores S. McCullough L. D. Cushman M. (2020). Identifying genetic and biological determinants of race-ethnic disparities in stroke in the United States. Stroke 51, 3417–3424. 10.1161/STROKEAHA.120.030425 33104469 PMC7594163

[B16] Miller E. T. King K. A. Miller R. Kleindorfer D. (2007). FAST stroke prevention educational program for middle school students: pilot study results. J. Neurosci. Nurs. 39, 236–242. 10.1097/01376517-200708000-00009 17847672

[B17] Mills M. C. Rahal C. (2020). The GWAS Diversity Monitor tracks diversity by disease in real time. Nat. Genet. 52, 242–243. 10.1038/s41588-020-0580-y 32139905

[B18] Mishra A. Malik R. Hachiya T. Jürgenson T. Namba S. Posner D. C.. (2022). Publisher correction: stroke genetics informs drug discovery and risk prediction across ancestries. Nature 612:E7. 10.1038/s41586-022-05492-5 36376532 PMC9712088

[B19] O'Connor R. E. McGraw P. Edelsohn L. (1999). Thrombolytic therapy for acute ischemic stroke: why the majority of patients remain ineligible for treatment. Ann. Emerg. Med. 33, 9–14. 10.1016/S0196-0644(99)70411-7 9867881

[B20] Park J. H. Ovbiagele B. (2016). Association of black race with recurrent stroke risk. J. Neurol. Sci. 365, 203–206. 10.1016/j.jns.2016.04.012 27206907 PMC4876018

[B21] Prapiadou S. Demel S. L. Hyacinth H. I. (2021). Genetic and genomic epidemiology of stroke in people of African ancestry. Genes 12:111825. 10.3390/genes12111825 34828431 PMC8619587

[B22] Radomski M. W. Palmer R. M. Moncada S. (1987). Endogenous nitric oxide inhibits human platelet adhesion to vascular endothelium. Lancet 2, 1057–1058. 10.1016/S0140-6736(87)91481-4 2889967

[B23] Radomski M. W. Palmer R. M. Moncada S. (1990). Characterization of the L-arginine:nitric oxide pathway in human platelets. Br. J. Pharmacol. 101, 325–328. 10.1111/j.1476-5381.1990.tb12709.x 1701676 PMC1917694

[B24] Robinson D. J. Stanton R. Sucharew H. Alwell K. Haverbusch M. Rosa F. D. R. L. (2022). Racial disparities in stroke recurrence: a population-based study. Neurology 99, e2464–e2473. 10.1212/WNL.0000000000201225 36041865 PMC9728039

[B25] Saini V. Guada L. Yavagal D. R. (2021). Global epidemiology of stroke and access to acute ischemic stroke interventions. Neurology 97(20 Suppl.2), S6–S16. 10.1212/WNL.0000000000012781 34785599

[B26] Skajaa N. Adelborg K. Horváth-Puhó E. Rothman K. J. Henderson V. W. Thygesen L. C.. (2022). Risks of stroke recurrence and mortality after first and recurrent strokes in Denmark: a nationwide registry study. Neurology 98, e329–e342. 10.1212/WNL.0000000000013118 34845054

[B27] Williams O. DeSorbo A. Noble J. Shaffer M. Gerin W. (2012). Long-term learning of stroke knowledge among children in a high-risk community. Neurology 79, 802–806. 10.1212/WNL.0b013e3182661f08 22875089 PMC3421152

[B28] World Health Organisation (2024). Stroke, Cerebrovascular Accident. Health Topics Web Site. World Health OrganisationEastern Mediterranean Region. Available online at: https://www.emro.who.int/health-topics/stroke-cerebrovascular-accident/index.html (accessed February 14, 2024).

